# Getting the full picture: Assessing the complementarity of citizen science and agency monitoring data

**DOI:** 10.1371/journal.pone.0188507

**Published:** 2017-12-06

**Authors:** Jeneen Hadj-Hammou, Steven Loiselle, Daniel Ophof, Ian Thornhill

**Affiliations:** 1 Earthwatch Institute (Europe), Mayfield House, Summertown, Oxford, United Kingdom; 2 Lancaster Environment Centre, Lancaster University, Lancaster, United Kingdom; 3 Department of Biotechnology, Chemistry and Pharmacy, University of Siena, Siena, Italy; 4 CoLA—Culture and Environment, Bath Spa University, Newton St. Loe, Bath, United Kingdom; INRA, FRANCE

## Abstract

While the role of citizen science in engaging the public and providing large-scale datasets has been demonstrated, the nature of and potential for this science to supplement environmental monitoring efforts by government agencies has not yet been fully explored. To this end, the present study investigates the complementarity of a citizen science programme to agency monitoring of water quality. The Environment Agency (EA) is the governmental public body responsible for, among other duties, managing and monitoring water quality and water resources in England. FreshWater Watch (FWW) is a global citizen science project that supports community monitoring of freshwater quality. FWW and EA data were assessed for their spatio-temporal complementarity by comparing the geographical and seasonal coverage of nitrate (N-NO_3_) sampling across the River Thames catchment by the respective campaigns between spring 2013 and winter 2015. The analysis reveals that FWW citizen science-collected data complements EA data by filling in both gaps in the spatial and temporal coverage as well as gaps in waterbody type and size. In addition, partial spatio-temporal overlap in sampling efforts by the two actors is discovered, but EA sampling is found to be more consistent than FWW sampling. Statistical analyses indicate that regardless of broader geographical overlap in sampling effort, FWW sampling sites are associated with a lower stream order and water bodies of smaller surface areas than EA sampling sites. FWW also samples more still-water body sites than the EA. As a possible result of such differences in sampling tendencies, nitrate concentrations, a measure of water quality, are lower for FWW sites than EA sites. These findings strongly indicate that citizen science has clear potential to complement agency monitoring efforts by generating information on freshwater ecosystems that would otherwise be under reported.

## 1. Introduction

Freshwater ecosystems are important for humans and wildlife, but they nevertheless face some of the most pressing environmental threats [1]. Rapid population growth, urbanisation and associated pollution have decreased the availability of good quality freshwater resources [2]. Despite this, there remains inadequate monitoring of freshwater systems world-wide and new approaches are required in order to identify changes in water quality over time and space [3]. Citizen science is rapidly emerging as one potential approach to complement standard monitoring programmes by contributing a larger extent and a finer grain data collection, both geographically and temporally [4–6].

Citizen science is “a form of research collaboration involving members of the public in scientific research projects to address real-world problems” [7]. Citizen-driven water quality monitoring projects are not uncommon. For example, Kinchy et al. [8] discussed the rise of such projects in New York and Pennsylvania as a response to an increase in natural gas drilling in the area. In another study, Lillesand [9] outlined the use of volunteer Secchi disk measurements to monitor the health of lakes, a project initiated by the local Department of Natural Resources. In addition to such cases where citizen science provides the sole source of data for government bodies, Loperfido et al. [10] present evidence where data collected through a community water monitoring programme in Iowa, IOWATER, improved the accuracy of the American Environmental Protection Agency freshwater site classifications. Similarly, Célleri et al. [11] showed how participatory monitoring efforts in the Andes were complimentary to the pre-existing official monitoring network; with citizen driven data collection operating on a high spatial but low temporal scales.

Studies have compared results obtained in citizen science projects to measurements made by professional researchers (e.g., [12]). While this is an important process which can contribute to an improved understanding of the error and bias in citizen science, citizen science and professional science do not necessarily need to be viewed as mutually exclusive [13]. Rather, the rise of citizen science can be seen as enabling a shift to a polycentric freshwater monitoring landscape. The availability and convergence of multiple information flows, whether from citizen science, remote sensing or in situ monitoring have major implications for the way water is governed [14].

In the European Union, water governance is strongly influenced by the Water Framework Directive (WFD) (2000/60/EC), a European Commission legislation that sets the standards for water quality and water resource management for EU member states. The WFD states a goal of attaining good ecological and chemical status of freshwater bodies [15]. In the UK, the environment agencies of England, Wales, Scotland, and Northern Ireland have been tasked with meeting the WFD’s legislative requirements and a UK Technical Advisory Group (UKTAG) was formed as a partnership between environment and conservation agencies working across the UK to provide technical advice towards WFD compliance [16]. While the WFD exists as an overarching framework with monitoring and assessment requirements, local governments ultimately have the flexibility to develop their own strategies to monitor and assess water bodies [17,18]. The UK has chosen to support a “Catchment Based Approach”. As such, river basin management plans, suggested by the WFD, seek to bring together various environmental stakeholders in a given catchment in an attempt to manage water collaboratively. While such management plans incorporate citizen and volunteer input, there is little discussion about the role of citizen science [19]. Thus, the opportunity exists for a restructuring of the existing freshwater monitoring network such that it integrates regulatory monitoring and new citizen science opportunities.

There have been few studies exploring the importance of citizen science to decision makers [20]. However, citizen science can provide multiple benefits to governments. With reference to the WFD, citizen science can be seen as a logical component to satisfy Article 14 regarding public participation as a mechanism for improving water quality throughout Europe [15]. Whitelaw et al. [21] also point out that citizen scientists can simply enable the expansion of an agency’s monitoring network while also helping to cut costs. In addition, Thornhill et al. [22] show that combining citizen science data with an environmental agency’s data improves data frequency by 50%.

As a citizen science project, FWW has engaged thousands of people in learning about and monitoring freshwater resources and has managed to amass over 17,000 water samples as of March, 2017. More importantly however, it has tailored data collection to allow specific research questions to be addressed [23]. Nevertheless, its ability to be used alongside other, more conventional and standardized environmental data collection has yet to be assessed.

This study seeks to contribute to a better understanding of the potential for citizen science to complement government agency monitoring efforts by comparing and contrasting the results of Environment Agency and citizen scientist (FreshWater Watch) field campaigns between spring 2013 and winter 2015 across the River Thames catchment. It is the first study to explore the spatiotemporal coverage and quantitative complementarity of agency and citizen science data. Through this comparison, a better sense of the potential role and value of citizen science to natural resource management is achieved.

## 2. Methods

### 2.1. Study site

**Fig 1:**
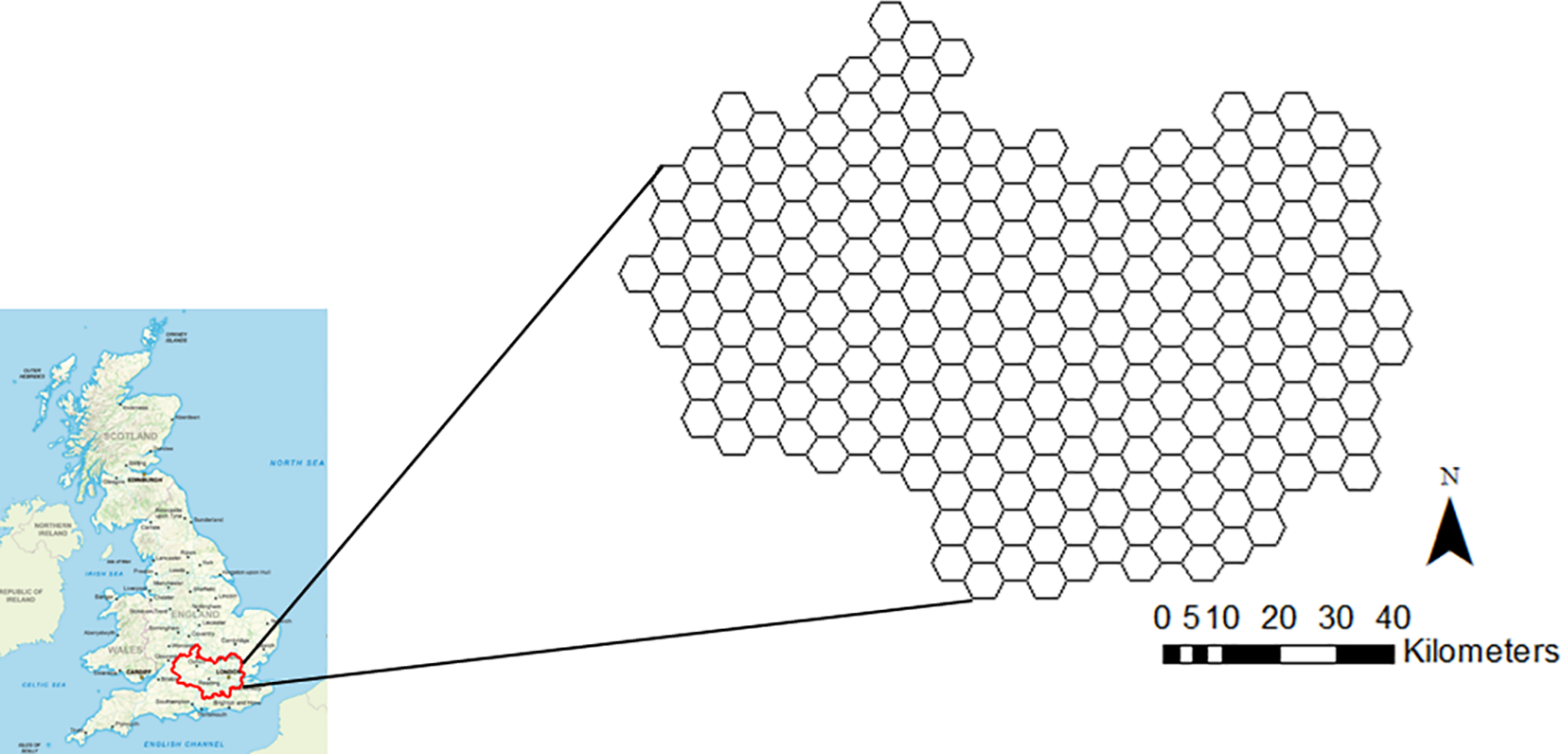
Thames catchment fitted with 5km hexagonal grid.

This study focusses on the Thames catchment (Fig 1) whose catchment boundaries were obtained from the online product, HydroBASINS [24]. The catchment is located in the southeast of England and has an area of 1,337,000 ha. Approximately 14.8 million people live within the catchment boundaries [25] which span the counties of Gloucestershire, Oxfordshire, Buckinghamshire, Hertfordshire, Surrey, Berkshire, Hampshire, Wiltshire and Greater London [26]. The dominant land-use type in the catchment is arable land. In the upper part of the catchment, arable land alternates with grassland, with approximately 17% of urban land coverage which increases in the southern and western sub-catchments, upstream from London [27]. The densest urban areas, encircled by suburbia, are in the centre east, represented by London and Greater London respectively [28,29].

### 2.2. Dataset acquisition and processing

FWW is Earthwatch’s global citizen science freshwater ecosystem research programme [22,30]. FWW citizen science participants in the Thames areainclude members of the general public active in wildlife and river associations, corporate volunteers, Earthwatch members, teachers and educators. Data were obtained by FreshWater Watchers, trained citizen scientists committed to making regular measurements (typically quarterly or monthly) in a local freshwater ecosystem of their choice and by Thames Water Blitz volunteers. Blitz volunteers consisted of local community members, contacted through various local outreach media and volunteer networks to participate in a single day monitoring event. All participants were trained in face-to-face training sessions or by viewing an online training video followed by a short quiz. Once trained, citizen scientists receive instructions on water quality sampling and data acquisition with their kit and have access to the FWW smartphone application with information on how to fill in a data sampling sheet with support from automated feedback. FWW data is available through the website https://freshwaterwatch.thewaterhub.org.

The EA is the governmental public body in the UK responsible for, among other duties, managing and monitoring water quality and water resources in England and Wales [31]. Selection of EA monitoring sites are objective-driven and based assessment units derived from natural features, geological changes (slope) and land use requirements (presence of potential pollution sources). In most cases, identification of water bodies as outlined by the WFD are considered [32].

Data on the water quality for regions covering the Thames catchment were acquired from the EA’s open access online platform available at http://environment.data.gov.uk/water-quality/view/landing. The data were filtered down to only include WFD compliance monitoring data and the determinand nitrate-N (N-NO_3_), a variable that could be directly comparable to FWW samples. Further to this, nitrate data were filtered down to include only samples taken between spring 2013 (1 March, 2013) and winter 2015 (31 December, 2015), a time-frame for which both the EA and FWW collected water quality measurements. Data for both actors were categorized into meteorological seasons to capture seasonal variations in water quality across a range of freshwater environments [33]. ArcGIS 10.3 was used to restrict geographical coverage to the Thames catchment.

### 2.3. Spatio-temporal complementarity of monitoring

A 5km hexagonal grid with 258 grid cells was used for map production and analysis of the three coverages: 1) the EA, 2) FWW, and 3) the overlap of the two (Fig 1). The 5 km scale was chosen because, globally, over 70% of the population lives within 5km of a freshwater body [34]. While this tessellation allowed for a comparison of measurement coverage and concentrations between data sources, it should be noted that this scale allows some overestimation of spatial overlap between datasets.

The hexagonal tessellation of the study area was constructed using the Machin 2007 tessellation tool because of its representational accuracy [35], and to reduce ambiguity of the nearest neighbour in assessing grid cell clustering [36]. Each grid cell was assigned a value depending on the number of seasons between spring 2013 and winter 2015 represented by the samples within it, either EA or FWW. Overlap between datasets was determined by comparing the seasons that were represented across the designated time period. Descriptive statistics explaining coverage of cells were produced in ArcGIS.

### 2.4. Running water sites: Stream-order

Running water sites were assigned a stream-order according to the Strahler [37] classification system determined using USGS HydroSHEDS flow accumulation and direction information with a 50 cell minimum accumulation condition. Using this template, the location of EA running water sites (already standardized as per EA sampling procedure) and FWW running water sites (determined by pooling points on the same stream within 50 m of each other and assigning them a standardized site name) were assigned a stream-order.

### 2.5. Still water sites: Size

The sizes (area in ha) of still water sites (ponds, lakes, reservoirs) sampled by the EA and FWW were determined by calculating the area of polygons outlining freshwater sites using OpenStreetMap [38]. Where this was not possible, site polygons were created by manually outlining the boundaries of the freshwater bodies, based on the OS (2016) open raster layer available through ESRI. Sites were then sorted into a <8ha category and a >8ha category, based on the Ramsar approach to distinguish lakes (>8ha) from ponds (<8ha) (Ramsar, 2009).

### 2.6. All sites: Nitrate concentration category

Nitrate concentrations for EA and FWW sites were compared to determine if water quality measurements for the two actors were similar. Nitrate was selected out of all the priority determinands from the datasets because it was the most directly comparable between the EA and FWW, considering the different methodology used by the two groups.

The test kit used by FWW produces a categorical classification for a sample’s nitrate concentration using colorimetric methods, while the EA uses standard laboratory methods to produces a continuous measurement. For FWW nitrate was measured using a naphthylethylenediamine method (Griess reagent) [39,40] in seven specific ranges from 0.2 to 0.5, 1, 2, 5 and 10.0 mg/L. Field methods were tested against laboratory methods (APHA, AWWA, WEF, 2012) using standard solutions and 1.5mL of natural water sample. Duplicate and triplicate measurements were made during training and quarterly quality control analysis. Variability between different citizen scientists in the same waterbodies (on the training days) was assessed. All data were cross-checked against specified criteria. If an inconsistent measurement was found, the citizen scientist who collected the dataset was notified and asked to confirm, delete or correct the measurement. In order to compare FWW and EA nitrate measurements, the nitrate measures taken for EA samples were placed into the same nitrate categories used by FWW. For sites with multiple measurements in a single season, a median nitrate concentration was calculated and then placed into a category. This category was then used to compare the conditions between different grid cells and data sources. The median number of samples in each seasonal grid cell was 1, making the use of an average more appropriate than the use of maximum concentration values and nearly identical to the use of the mode.

### 2.7. Statistical analysis

Standard statistical measurements were made to compare 1) the frequency (percentage) of EA and FWW sites with still water site areas <8 ha and areas >8 ha, 2) the distribution of the percentage of EA and FWW sites with stream-orders between 1 and 6, and 3) the distribution of the percentage of FWW and EA sites with average nitrate values in each category. Chi-squared tests of association were conducted to determine whether there was a relationship between: area of the still water sites and identity as an FWW or EA site, stream-order of the running water site and identity as an FWW or EA site, and nitrate category and identity as an EA or FWW site. Where the tests were significant, the effect size of correlation was calculated as the Cramer’s V statistic. In order to determine the categories for which the association was most significant, a post-hoc test was carried out comparing adjusted residuals [41]. All statistical analyses were performed using IBM SPSS Statistics 21.

## 3. Results

### 3.1. Spatio-temporal comparison

**Fig 2:**
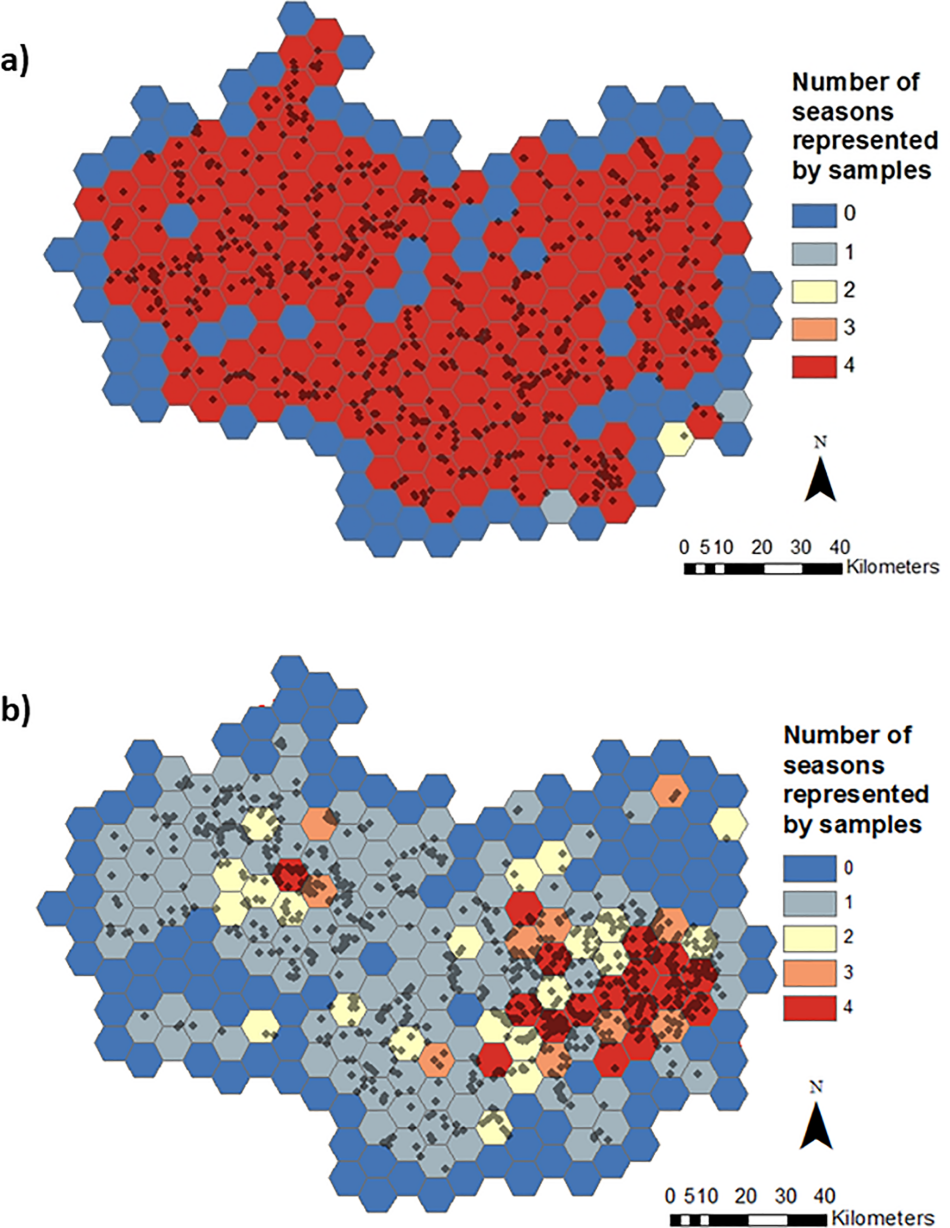
Thames basin divided into a 5km hexagon grids. The grid cells are coloured according to the number of seasons (0 = blue to all 4 = red) represented by nitrate samples shown as black dots collected by a) the EA and b) the FWW between spring 2013 and winter 2015.

**Fig 3:**
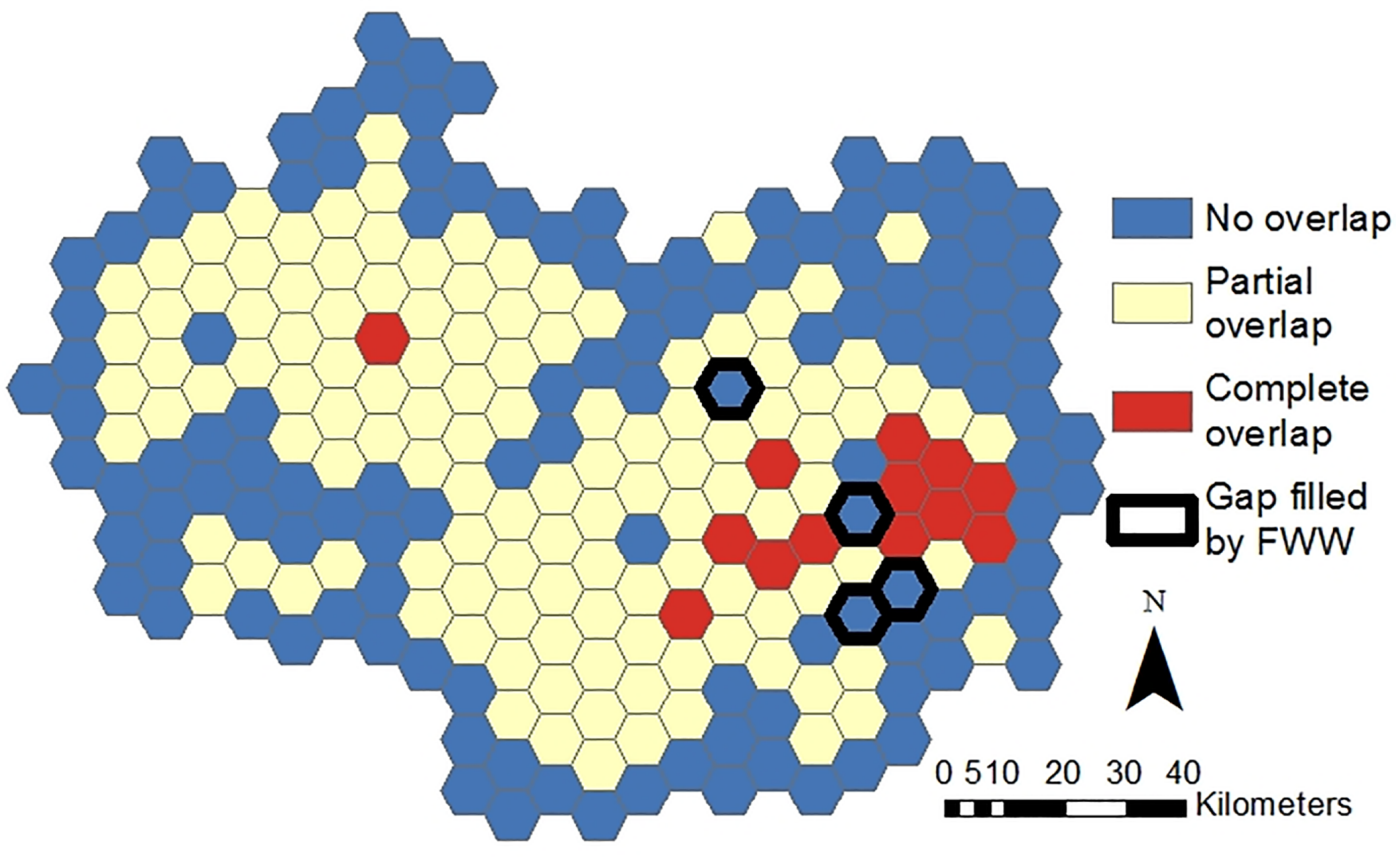
Overlap in grid cells sampled by the EA and FWW, where complete overlap (red) represents grid cells where both EA and FWW samples are represented by all the seasons, partial overlap (yellow) represents grid cells where EA and FWW are represented by samples collected in some of the same seasons, and no overlap (blue) represents grid cells where none of the same seasons are represented by the two datasets. Grid-cells where spatiotemporal gaps are filled by FWW samples (areas where FWW citizen scientists collected samples for all the seasons, but the EA did not) are represented by a black outline. The time period investigated is between spring 2013 and winter 2015.

The results of the spatio-temporal comparison of EA and FWW monitoring show that the two actors have different sampling habits, but there is still overlap in sampling effort, with gaps in EA sampling being filled by FWW activities. A higher temporal coverage of EA nitrate data was evident, with 68% of EA grid cells (Fig 2A) sampled in all four seasons, in comparison to 7% of grid cells for FWW (Fig 2B). The two actors had similar spatial coverage; FWW covered 60% of the grid cells (Fig 2B), while the EA covered 69% of the grid cells (Fig 2A). There was an overlap between datasets; with over half (52%) of the grid cells showing some or complete overlap between FWW and the EA (Fig 3). FWW was found to fill in some gaps in EA data collection effort, with 2% of grid cells being sampled in all four seasons by FWW but not by the EA (Fig 3).

### 3.2. Still water sites

**Fig 4:**
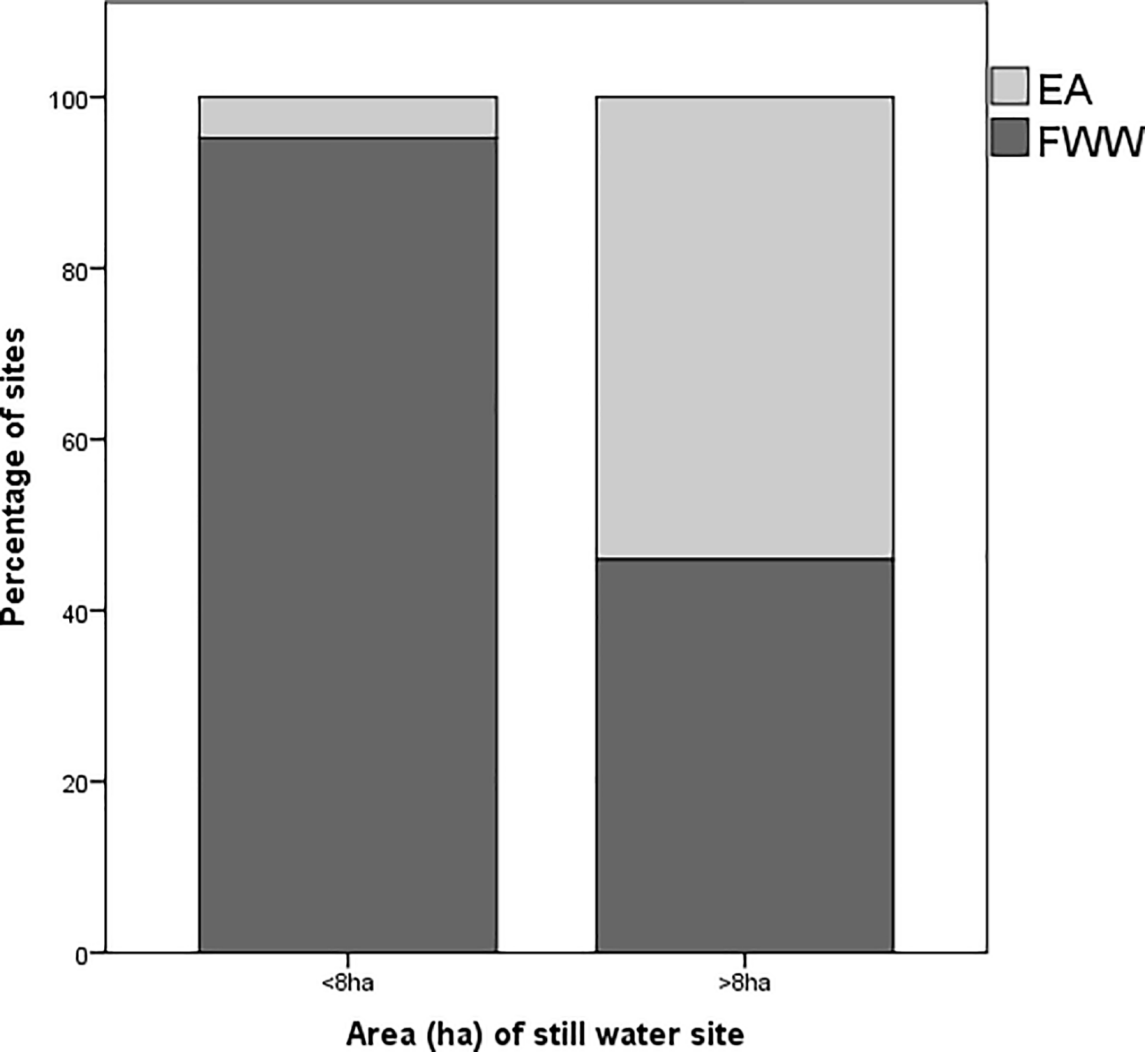
The percentage of still-water sites (ponds, lakes, wetlands) sampled by the Environment Agency (EA) (N = 25) and Freshwater Watch (FWW) (N = 156) citizen scientists with areas less than 8 ha and greater than 8 ha. EA sites are represented by a light grey colour and FWW sites are represented by a dark grey colour.

To assess whether the types of ecosystems being sampled by the EA and FWW are complementary, the sizes of the still water sites sampled by the two actors were compared. There was a significant association between the area of the still water body (<8 ha or >8 ha) and its origin as either a FWW or EA site (x^2^ = 56.95, p<0.001; Cramer’s V = 0.558, p<0.001). A significantly greater proportion (95%) of FWW sites had a dimension of <8ha (Adjusted-residual post-hoc Bonferroni, p<0.001). Conversely, a significantly greater proportion (54%) of the EA sites were >8ha (Adjusted-residual post-hoc Bonferroni, p<0.001). The median area of FWW still water body sites, 0.38 ha, was smaller than the median area of EA still water sites, 19.07 ha (Mann-Whitney U = 513, n_2_ = 156, n_1_ = 25, p<0.001) (Fig 4).

### 3.3. Running water sites

**Fig 5:**
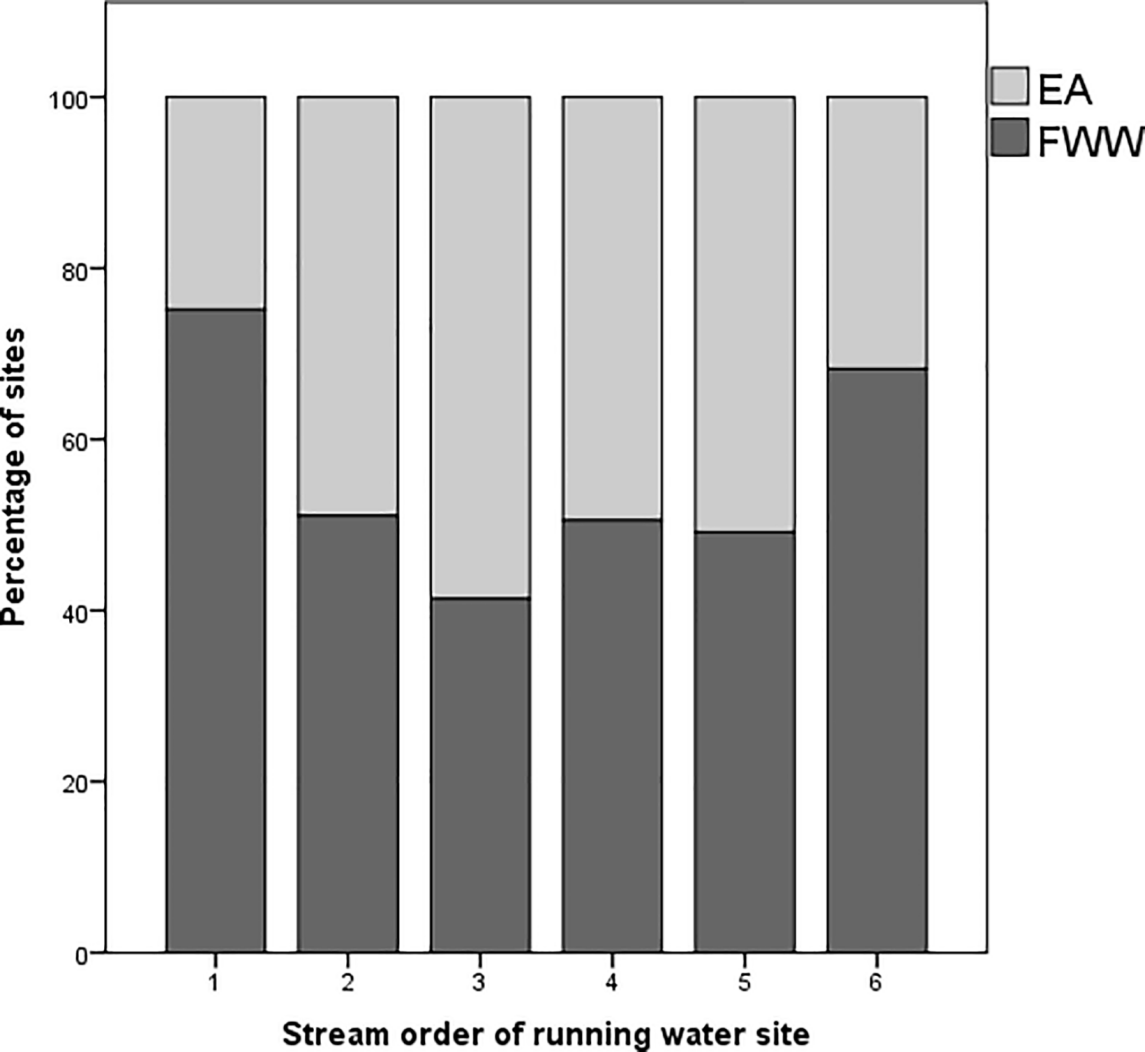
Stacked bar-chart showing the percentage of EA running water sites (N = 666) and FWW running water sites (N = 714) within each stream-order. EA sites are represented by a light grey colour and FWW sites are represented by a dark grey colour.

Similar to the comparison of the size of still water sites, the complementarity between the scales and character of the sampling efforts by the EA and FWW to monitor running water sites was determined using stream-order. There was a significant association between the stream-order of the site and its origin as either an EA or FWW site (X^2^ = 69.66, p<0.001; Cramer’s V = 0.225, p<0.001). FWW had a significantly higher proportion of sites (75%) in stream-order 1 (Adjusted-residual post-hoc Bonferroni, p<0.001) and stream-order 6 (68%) (Adjusted-residual post-hoc Bonferroni, p = 0.02). Conversely, the EA had a significantly greater proportion of sites (59%) in stream-order 3 (Adjusted-residual post-hoc Bonferroni, p<0.001) (Fig 5).

### 3.4. Nitrate comparison

**Fig 6:**
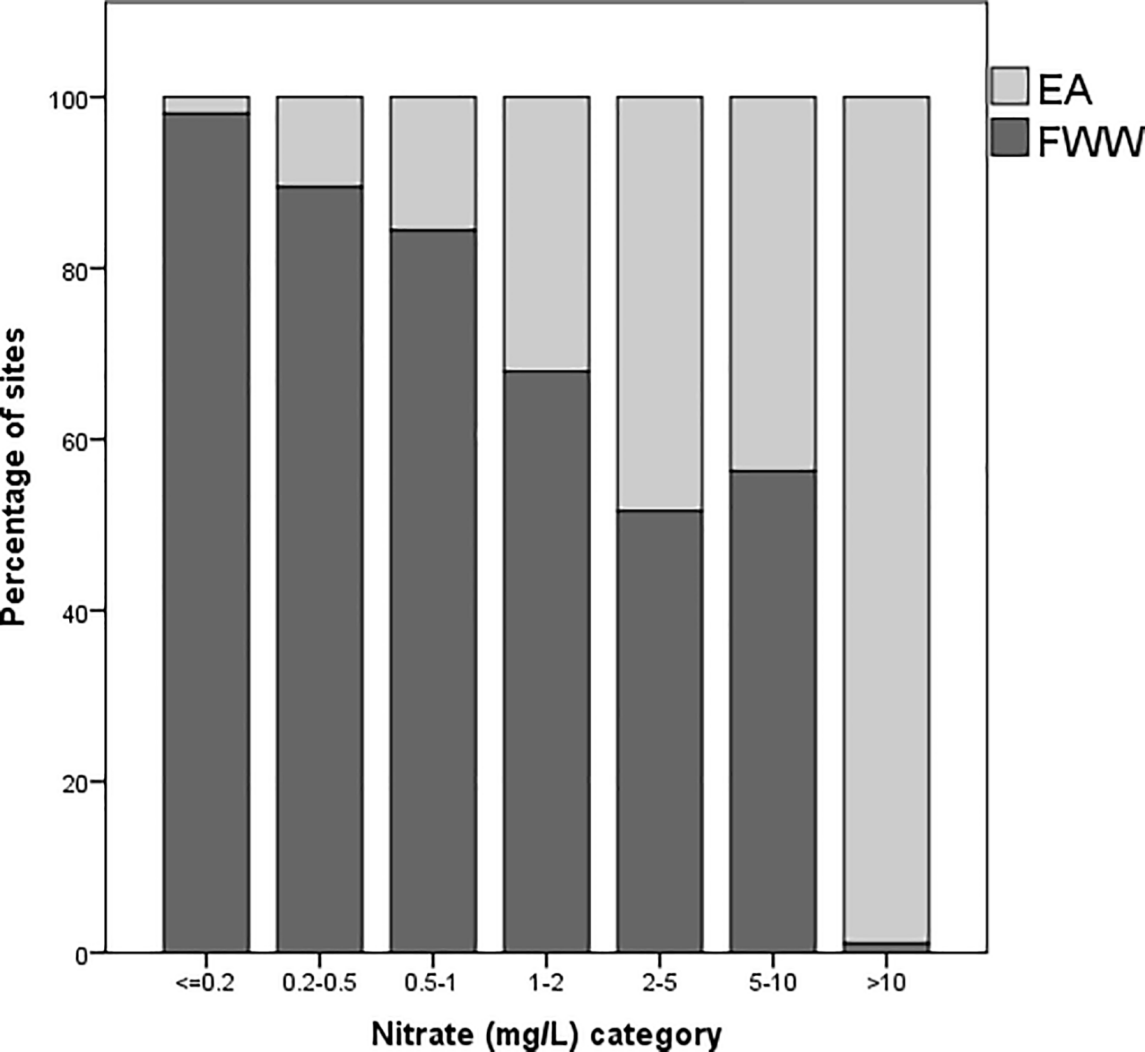
Stacked bar-chart showing the percentage of sites (still-water and running-water) with an average nitrate value within each nitrate category, where N = 2 for EA still-water sites with nitrate samples, N = 613 for EA running-water sites with nitrate samples, N = 156 for FWW still-water sites with nitrate samples, and N = 714 for FWW running-water sites with nitrate samples. EA sites are represented by a light grey colour and FWW sites are represented by a dark grey colour.

When EA and FWW site nitrate water quality measurements were compared, a significant association between average nitrate category (mg/L) and its origin as an EA or FWW site was found (x^2^ = 259.67, p<0.001; Cramer’s V = 0.418, p<0.001). A significantly greater proportion of FWW sites than EA sites were associated with the lower nitrate categories “< = 0.2” (98%) (Adjusted-residual post-hoc Bonferroni, p<0.001), “0.2–0.5” (89%) (Adjusted-residual post-hoc Bonferroni, p<0.001), and “0.5–1” (84%) (Adjusted-residual post-hoc Bonferroni, p<0.001). A significantly greater proportion of EA sites than FWW sites are associated with the higher nitrate category “>10” (99%) (Adjusted-residual post-hoc Bonferroni, p<0.001) (Fig 6).

## 4. Discussion

The transformative role of citizen science to scientific data collection is a phenomenon that is widely referenced [42–45]. Delaney et al. [46] refer to citizen scientists as the “key solution” to a more comprehensive monitoring network; they go to the areas that scientists do not monitor themselves. While citizen science has been proposed as a neoliberal alternative to agency monitoring, enabling governments to cut costs and responsibility to ensure healthy ecosystems [47,48], this study demonstrates that it is the complementary nature of the relationship that provides the best results.

### 4.1. Spatio-temporal analysis

The EA monitoring programme covers the majority of catchments across all the seasons (Fig 2A), following its role as a regulatory agency of the national government body, required to sample each of their 7000 predefined sites 12 times a year [49]. On the other hand, while FWW also covers a large proportion of the Thames catchment geographically, it does not sample sites across all four seasons in a uniform manner (Fig 2B). This finding consolidates conclusions made by Thornhill et al. [22] regarding the tendency for FWW citizen scientists to sample sites mostly in the spring and summer, despite the programme request to “visit each of the chosen water bodies at least four times per year, or once every three months” [23]. This temporal sampling bias has also been found in other citizen science projects [50,51]. Such a bias could influence conclusions drawn about the status of water bodies as water quality varies across the seasons [52,53]. This is particularly important for smaller waterbodies, e.g. some ponds are only seasonal; with a seasonal bias in sampling effort, seasonal ponds might not be represented in the dataset, but they are likely to have different characteristics to other ponds that are represented [54]. This could therefore skew water quality data. The importance of this bias depends on the purpose of monitoring; if citizen scientists consistently sample water bodies in the spring or summer, then an analysis of summer trends over the years could still be completed. The resolution and reliability of EA data is however essential to assess change for shorter time periods. The similarly large geographical coverage of both FWW and the EA highlights the potential usefulness of the data. Tulloch et al. [55] show that in the case of atlases based on volunteer observations, the more coverage a dataset has, the more it gets used in research. The vast coverage of FWW data points is a result of the UK project design, which allows participants to monitor sites of their choice, and is concurrent with other research suggesting that citizen science tends to be characterized by large geographical extents [56].

EA and FWW data spatially overlap in over half of the study area (Fig 3), indicating a possible redundancy in data generation. Redundancy can be both positive and negative. Buytaert et al. [14] state that redundancy could make a monitoring network more “robust”. Similarly, Connors et al. [57] describe the inherent redundancy of citizen science as a mechanism for “peer-reviewing” and “self-correcting”. On the other hand, from an economic or volunteer use perspective, the costs of over-sampling the same areas could be considered inefficient.

The areas of sampling overlap tend to be in the urban and suburban areas in and around London. Such areas might be receiving more attention from citizen scientists because they are closer to where they live and work. In order to facilitate the ease and therefore frequency of water quality monitoring, the FWW website encourages volunteers to sample water “at locations near [their] home or work–ideally within or on the edge of an urban area” [23]. The other area of overlap is in Oxford, where the Earthwatch office is located, and where the strongest outreach and volunteer network is present.

The spatial distribution of EA monitoring sites across the seasons is more widespread (Fig 2B). The EA selects site locations in accordance with recommendations from the UK-TAG on the WFD [58]. The aim of authorities working under the WFD is to establish as many water quality monitoring stations as necessary within a river basin district so that an “overview of water status has an acceptable level of confidence and precision” [59]. The EA therefore has a legal obligation to monitor water bodies across the catchment, and not only those that are conveniently accessed like FWW volunteers. Nevertheless there were a number of grid cells where monitoring was limited to FWW activities, falling into clusters of frequently monitored cells, again in the east of the catchment. With an area of 65 km^2^ each, their cumulative value is large. This “gap fill[ing]” by citizen scientists is often referenced as an important contributor to attaining a more complete and holistic understanding of the total environment [5,60]. Here, an element of complementarity is introduced, whereby the citizen science project enhances the environmental monitoring network which is the central responsibility is the national environmental agency. Some authors have suggested that citizen science could serve as an early warning system providing initial monitoring data to better direct professional monitoring [61]. However, in the case of FWW gap filling in the Thames catchment, a more equally complementary approach between volunteer monitoring and agency monitoring is demonstrated. FWW data supplements the EA network that has already been established. Tulloch et al. [55] highlight that in the opposite circumstance where volunteers are the foundations of a monitoring programme, targeted professional sampling will then be required to reduce bias and unevenly distributed effort. However, if data from multiple sources is pooled or integrated, bias acting in various directions can be reduced as statistical power increases [62].

### 4.2. Still water sites

Taking a finer scale, ecological look at the data, FWW activities obtained more information on still water sites than the EA, and FWW tends to sample smaller freshwater bodies <8ha (Fig 4). According to Biggs et al. [63], small water bodies including ponds and low-order streams often represent the healthiest and most ubiquitous freshwater bodies. They tend to support a wide range of unique biodiversity, more so than larger still waterbodies or running waters [64]. Nevertheless, small waterbodies are also the least studied freshwater source and have largely been overlooked by EU and UK legislation [63,65], even though they represent the largest number of still water bodies and a land area similar to the largest lakes [66]. Citizen scientists can therefore play an important role in collecting information to understand these vital but poorly studied ecosystems. A report produced by the European Environmental Bureau suggests that an appreciation of the necessity and the drive to be more inclusive of small waterbodies in monitoring efforts is present, but the delivery of action is still lacking [67].

Citizen scientists’ preference to sample small waterbodies more frequently than large waterbodies might have multiple causes, the most obvious of which is ease of access and abundance. Boakes et al. [50] assessed behavioural patterns of volunteer recording activity in citizen science projects and found that unique characteristics and identified hotspots make a site more likely to be visited. In the case of water quality monitoring, small waterbodies might be considered a hotspot, due to their increased visibility. This has implications for the future development of monitoring networks. An understanding of where citizen scientists are more likely to measure would ensure that regulatory efforts are more targeted to areas that are likely to be underrepresented by citizen scientists, considering most regulatory monitoring networks have resource limitations [68].

### 4.3. Running water sites

Similar to the results found for still waterbodies, FWW citizen scientists tended to favour sampling small first-order streams. However, they also sampled sixth-order waterbodies frequently (Fig 5). Again, accessibility and proximity are likely to be contributing factors. In addition, the cultural importance of the waterbody might play a role in its selection. The Thames River is an important stream-order six water body and a prominent feature of the landscape across all county boundaries [69]. The high cultural value and significance could be what is driving citizen scientists to test its water quality. Members of community-based monitoring projects often have an increased sense of environmental stewardship and connection to culturally valuable assets of a landscape [70]. The same principle might extend to volunteers involved in a global monitoring network; each individual member could be drawn to the site they feel most connected with, be it a small stream behind their house, or the large river that characterises their city. Further research to assess the relative importance of cultural value and accessibility in citizen science would help in the design of future programmes.

The results also show that the EA sampled more stream-order 3 sites than FWW (Fig 5). The tendency for the EA and FWW to monitor sites of some stream-orders more than others indicates that neither actor is sampling sites evenly across stream-orders in the Thames region. This bias, which has been used to criticize the value of citizen science [71], can therefore be overcome with an integrated monitoring network approach. Such an approach would address the bias present when any one given actor is made responsible for all monitoring.

### 4.4. Nitrate concentrations

When the distribution of nitrate categories for EA and FWW sites was compared, the results showed that FWW sites were more associated with lower concentration categories compared to EA sites, which had a large number of sites with high nitrate concentrations (>10 mg/L) (Fig 6). Thus, neither the EA nor FWW have an even spread of sites across nitrate concentrations, and moreover, show opposite tendencies for association. One possible explanation for this difference between the EA and FWW water quality measurements might be that the methods used for sampling and measurement were different. Indeed, some citizen science water quality measurements have been shown to not accurately represent water quality compared to agency lab-tested measurements [72]. However, FWW methods were designed to obtain high quality, scientifically useful data with an appropriate quality control of the methods and data acquired [30]. Quality control in each of the 30 global FWW projects shows that the trends and distributions of nitrate concentrations follow those made in laboratory and side by side studies [73,74].

Given the above, the differences in nitrate data between the EA and FWW are indicative of differences in the types of water bodies being sampled by the two actors. Lotic and lentic habitats have different water chemistry and have typically different catchment sizes [75]. Thus, because FWW has a considerably greater number of still water sites than the EA, the results confirm that still waterbodies, particularly small still waterbodies, have lower nitrate concentrations [74]. Furthermore, a large proportion of FWW sites are of stream-order 1, while the EA has most sites in a stream-order 3. Montreuil et al. [76] found that their study sites of stream-order 6 had mean nitrate concentrations 47% lower than stream-order 2 and 3 streams. Thus, the difference in nitrate concentrations is likely due to the size and characteristics of the stream catchment as well as the ecology of the waterbodies being sampled.

## 5. Conclusion

This study shows how citizen science can support polycentric water governance by enhancing agency monitoring through gap filling, both spatially and ecologically. FWW volunteers were found to monitor different types and sizes of waterbodies to the EA with similar geographical coverage, but less uniform temporal coverage. Citizen scientists monitored a greater proportion of still water sites <8ha and more 1^st^ order streams than the EA. They also monitored more still water sites overall. The European Environment Agency recommends stratified sampling when designing a river monitoring network, so that more balanced information on small, medium and large rivers can be obtained. There are obvious limitations for most regulatory monitoring, which can lead to conflicts in meeting site-specific (potential pollution sources), priority habitat and uniformity objectives. The inclusion of citizen science acquired information may help to meet multiple objectives [77].

In the present comparison, there was a clear difference in the nitrate concentration distributions between the two campaigns. This was at least partially related to the type of waterbodies monitored, in particular the increased attention by citizen scientists to small, still waterbodies, thereby supporting the studies that show that these waterbodies are less impacted than large running waterbodies. Moreover, small waterbodies have often been neglected by monitoring agencies. By including citizen scientist derived data, essential information on their status over time can be collected, potentially serving as a driver for more inclusive monitoring by networks in the future.

The UK’s catchment based approach to the management of freshwater resources provides a good foundation for polycentric water governance and inclusivity. By building partnerships between stakeholders within catchments, the various aims and monitoring efforts of individual organisations can be better aligned. Additionally, river basin management plans can incorporate a wider evidence base and reach out to the broad citizen science network for input.

Future work is needed to further understand the drivers in behaviour of citizen scientists involved in water quality monitoring, in order to better target efforts of various actors in a given network. This study should serve as encouragement for citizen science projects; if designed with the purpose of addressing scientific research questions, as with the FWW programme, citizen science can provide insights into the state of the environment unmonitored by government agencies. Such data collection efforts can be encouraged by agencies and also serve to highlight where monitoring needs to be improved. At present however, citizen science programmes like FWW have proven to be beneficial in multiple ways and could contribute to tackling the major freshwater problems faced in our growing urban and suburban environments.
